# Exploring Connections Between Mental Health, Burnout, and Academic Factors Among Medical Students at an Iranian University: Cross-Sectional Questionnaire Study

**DOI:** 10.2196/58008

**Published:** 2025-05-15

**Authors:** Elham Faghihzadeh, Ali Eghtesad, Muhammad Fawad, Xiaolin Xu

**Affiliations:** 1 Department of Biostatistics and Epidemiology, Zanjan University of Medical Sciences, Zanjan, Iran; 2 School of Medicine, Zanjan University of Medical Sciences, Zanjan, Iran; 3 School of Public Health, The Second Affiliated Hospital, Zhejiang University School of Medicine, 866 Yuhangtang Road, Hangzhou, Zhejiang, 310058, China

**Keywords:** emotional exhaustion, exhaustion, cynicism, academic efficacy, burnout, physician burnout, mental health, mental illness, mental disease, mental disorder, medical education, medical knowledge, medical training, medical student, resident physician, resident doctor, residency, residency training

## Abstract

**Background:**

Medical students face high levels of burnout and mental health issues during training. Understanding associated factors can inform supportive interventions.

**Objective:**

This study aimed to examine burnout, psychological well-being, and related demographics among Iranian medical students.

**Methods:**

A cross-sectional survey was conducted among 131 medical students at an Iranian University. The instruments used included the Maslach Burnout Inventory-Student Survey and the Symptom Checklist-90-Revised. Descriptive statistics, multivariate regression, and tests for group differences were used to analyze the data.

**Results:**

The MBI-SS subscale scores indicated moderate emotional exhaustion, mean 15.00 (SD 7.08) and academic efficacy, mean 14.98 (SD 6.29), with lower cynicism, mean 10.85 (SD 5.89). The most commonly reported mental health issues were depression and obsessive-compulsive disorder. Poor psychological well-being was associated with higher overall burnout, but no significant gender differences were found. Burnout levels varied by academic year across all Maslach Burnout Inventory-Student Survey domains.

**Conclusions:**

Despite their health education, medical students in this study reported significant burnout and mental health distress, with strong associations between the two. These issues may impact student retention and post-graduation practice plans. Supporting well-being during training is critical for positive student and physician outcomes.

## Introduction

Kary and Pines [1] initially posited the concept of academic tedium and its impact on students. They suggested that this phenomenon is not confined to a specific educational level but can manifest at various stages of schooling, including both school and university environments [1]. Based on the author’s viewpoint, students might be struggling with a condition marked by a fading enthusiasm for learning, noticeable lack of motivation, and an overwhelming sense of emotional exhaustion. Later, Maslach and Jackson [2,3] specified burnout as the experience of physical and emotional drain caused by chronic stress. Burnout is the state of physical and mental fatigue caused by work, study, or any caregiving activities. It can also be known as an adverse emotional, cognitive, and physical reaction to the study, work, and life pressures. Burnout was officially classified by the World Health Organization as an occupational phenomenon in 2019 and included in the International Classification of Diseases (*ICD-11*).

Educational burnout is a type of burnout experienced during studying. To better view educational burnout, it was expanded to three factors: emotional exhaustion, cynicism, and feelings of inefficacy [4,5]. Emotional exhaustion reflects feelings of exceeding emotional resources due to academic demands. Cynicism is a negative, unresponsive, or overly snapped response to a phenomenon. Feelings of inefficacy refer to a reduction in academic effort, leading to a sense of incompetence and reduced academic achievement. Based on findings from a systematic review published in 2021, it was determined that educational burnout affected more than 40% of students [6]. This outcome implies a heightened susceptibility to burnout among medical students on a global scale. However, few studies have examined this problem, specifically among medical students in Iran. While 16% of Iranian medical students reported burnout in one study [7], assessing prevalence rates at individual universities could further inform supportive programs. Educational burnout has an essential role in medical students’ overall health and could easily impact the quality of their learning [8].

A study on 14,000 students from different countries showed that approximately 35% of the students had been diagnosed with at least one mental health disorder, such as depression or anxiety [9]. Among students, university students showed a higher likelihood of mental health disorders, and among them, medical students’ issues were significant. Medical schools pose multiple demands on students. First, enrollment in medical training coincides with adolescence and early adulthood, periods already associated with vulnerability to mental health disorders [10-12]. Second, the intense nature of medical education requires students to assimilate vast amounts of health information while coping with exposure to myriad diseases [11,12]. Consequently, studies report substantial rates of depression (11%‐37%), anxiety (7.4%‐30%), and other issues in this population internationally [13-15]. Evidence suggests that positive mental health aids coping [16], yet remains understudied in Iranian cultures.

Extensive evidence demonstrates intricate connections between burnout and mental health issues among medical students. Additional studies reveal substantially higher risks of depression, anxiety, suicidal ideation, concentration deficits, and physical symptoms compared to their peers [12,17-20]. Up to half of graduating students experience burnout, linking this syndrome to exacerbated mental health decline [18]. Ultimately, these concerning rates significantly exceed general population trends, underscoring the crisis of psychological well-being in medical education. Implementing supportive interventions requires further investigating specific student populations.

The aims of this study are twofold. Primarily, we assess the prevalence of mental health issues and burnout among native Iranian medical students at Zanjan University of Medical Sciences. Additionally, we delineate connections between mental health status and burnout risk by evaluating associated academic and personal factors. By understanding these relationships, targeted interventions can eventually be developed to promote the psychological well-being of Iran’s future physicians during their demanding training period.

## Methods

### Study Design and Participants

This cross-sectional study was conducted at Zanjan University of Medical Sciences, Zanjan, Iran, focusing on the experiences of 1500 medical students. These trainees constituted the target of our research, with their perspectives and characteristics as students comprising the central subject of investigation. Participants were recruited using a convenience sampling method. Our research team directly contacted the students, explained the study’s aims, invited their voluntary participation, and emphasized the confidentiality of their responses. We then sent an electronic survey link to consenting participants. Strict data quality control measures were implemented, with incomplete questionnaire submissions excluded from the analysis to uphold the integrity of the results. Based on a previous study [21], the minimum required sample size was 120 students; however, 140 surveys were distributed, and 131 fully completed questionnaires were returned. Those with missing data or students who indicated having a diagnosed mental health issue were excluded.

### Measures

#### Demographics

The basic sociodemographic information included age, sex, residence, history of a positive COVID-19 test, underlying diseases, diagnosis of mental health issues, and level of education. The levels of education were categorized into three sections: the initial seven semesters, referred to as preclinical, followed by a two-semester externship, and finally, a three-semester internship. At the preclinical level, students learned about basic sciences and pathophysiology; in the externship phase, they would pass a short course in each hospital unit. The residential status comprises a parental home, independent home, or a dormitory. Students were asked directly about underlying diseases, including diabetes, hypertension, and chronic disease. Additionally, they were asked whether they had been diagnosed with any mental health condition and whether they had received any treatment.

#### Burnout Measurement

Burnout symptoms were measured by the Persian version of the MBI-SS [3,22,23]. It comprises 15 items, which are divided into three dimensions: emotional exhaustion, cynicism, and academic efficacy . Each item has been rated on a 7-pointed Likert scale. Academic efficacy scores were reverse-coded; therefore, it was scored oppositely. A high score in three dimensions indicated greater burnout. The maximum possible scores for emotional exhaustion, cynicism, and academic efficacy were 30, 24, and 36, respectively.

#### Mental Health Measurement

The Symptom Checklist 90 (SCL-90), developed by Derogatis was used to assess mental health. This scale consists of 90 items, each rated on a 5-point Likert scale, effectively measuring ten primary psychological symptoms [24]. The ten psychological symptoms measured by the Symptom Checklist-90-Revised (SCL-90-R) are somatization, obsessive-compulsive, interpersonal sensitivity, depression, anxiety, hostility, phobic anxiety, paranoid ideation, psychoticism, and sleep problems. If a person’s average score for the questions related to these symptoms was greater than 2, it indicated potential psychological issues. The Global Severity Index (GSI) was calculated for our analysis, which measures the extent or depth of psychiatric disturbances. Specifically, the GSI is the average score across all responded items and serves as an overall measure of psychiatric distress. Therefore, this study analyzed the positive rate of each subscale and the GSI. Notably, the validated Persian version of the SCL-90-R was used for this student population [25].

### Statistical Analysis

We performed all statistical analyses using SPSS (version 20.0; IBM Corp) and Stata (version 12; StataCorp), and figures were drawn using R software (version 4.4.1; R Foundation for Statistical Computing) was used for visualization, including the ggplot2 package (version 3.5.1). Multivariable regression was used to assess factors associated with the three burnout subscales to examine the correlation between the response variables. The model included sociodemographic variables such as history of COVID-19, place of residence, and mental health status. The variables that were found to have a significant impact on the outcome were retained in the model.

### Ethical Considerations

Ethics approval for this study was provided by the Ethics Committee of Zanjan University of Medical Sciences (IR.ZUMS.REC.1400.418). This committee approved all experimental protocols. The authors confirmed that relevant guidelines and regulations were used in all experiments. No participants were younger than 16 years. All students provided written informed consent.

## Results

The study initially involved 140 students; after excluding those who dropped out due to missing answers or diagnosed mental illness, the remaining sample consisted of 131 participants. The average age of these remaining participants was approximately 24 years, with a mean of 23.95 (SD 3.69) years. Table 1 summarizes other sociodemographic characteristics of the student group. Approximately 66% were female students, while only 10% of the participants were married. An almost equal percentage of students across different academic levels completed the questionnaires. Additionally, 62% of the students had a history of a positive COVID-19 test, while 3.8% reported underlying diseases.

**Table 1.** Socio-demographic characteristics.

Sociodemographic variables	Participants (N=131), n (%)
Sex	
Male	44 (33.6)
Female	87 (66.4)
Marital status	
Single	118 (90.1)
Married	13 (9.9)
Residence	
Parental home	53 (40.5)
Own home	43 (32.8)
Dormitory	35 (26.7)
Positive COVID-19 test history	
No	50 (38.2)
Yes	81 (61.8)
Underlying diseases	
No	126 (96.2)
Yes	5 (3.8)
Academic level	
Preclinical	42 (32.1)
Externship	47 (35.9)
Internship	42 (32.1)

Table 2 shows the positive rates of SCL-90-R subscales by sex. Obsessive-compulsive disorder and depression showed the highest prevalence among symptoms. A *χ*^2^ test examined the percentage differences between male and female students. The only symptom found to be statistically significant between the two sex was phobic anxiety. Among female students, paranoid ideation had the highest prevalence, whereas obsessive-compulsive disorder was more prevalent among male students.

**Table 2.** Comparison of SCL-90-R^a^ subscales based on sex.

	SCL-90-R positive rates in male students n (%)	SCL-90-R positive rates in female students, n (%)	Total SCL-90-R positive rate, n (%)
Hostility	10 (22.7)	13 (14.9)	23 (17.6)
Anxiety	11 (25.0)	14 (16.1)	25 (19.1)
Obsessive-compulsive disorder	14 (31.8)	18 (20.7)	32 (24.4)
Interpersonal sensitivity	10 (22.7)	19 (21.8)	29 (22.1)
Somatization	6 (13.6)	12 (13.8)	18 (13.7)
Psychoticism	5 (11.4)	7 (8.0)	12 (9.2)
Paranoid ideation	8 (18.2)	21 (24.1)	29 (22.1)
Depression	12 (27.3)	20 (23.0)	32 (24.4)
Phobic anxiety^b^	9 (20.5)	7 (8.0)	16 (12.2)
Others	8 (18.2)	11 (12.6)	19 (14.5)

a SCL-90-R: Symptom Checklist-90-Revised.

b *P* value (*χ*2 test)=.04 male versus female.

**Figure 1.**
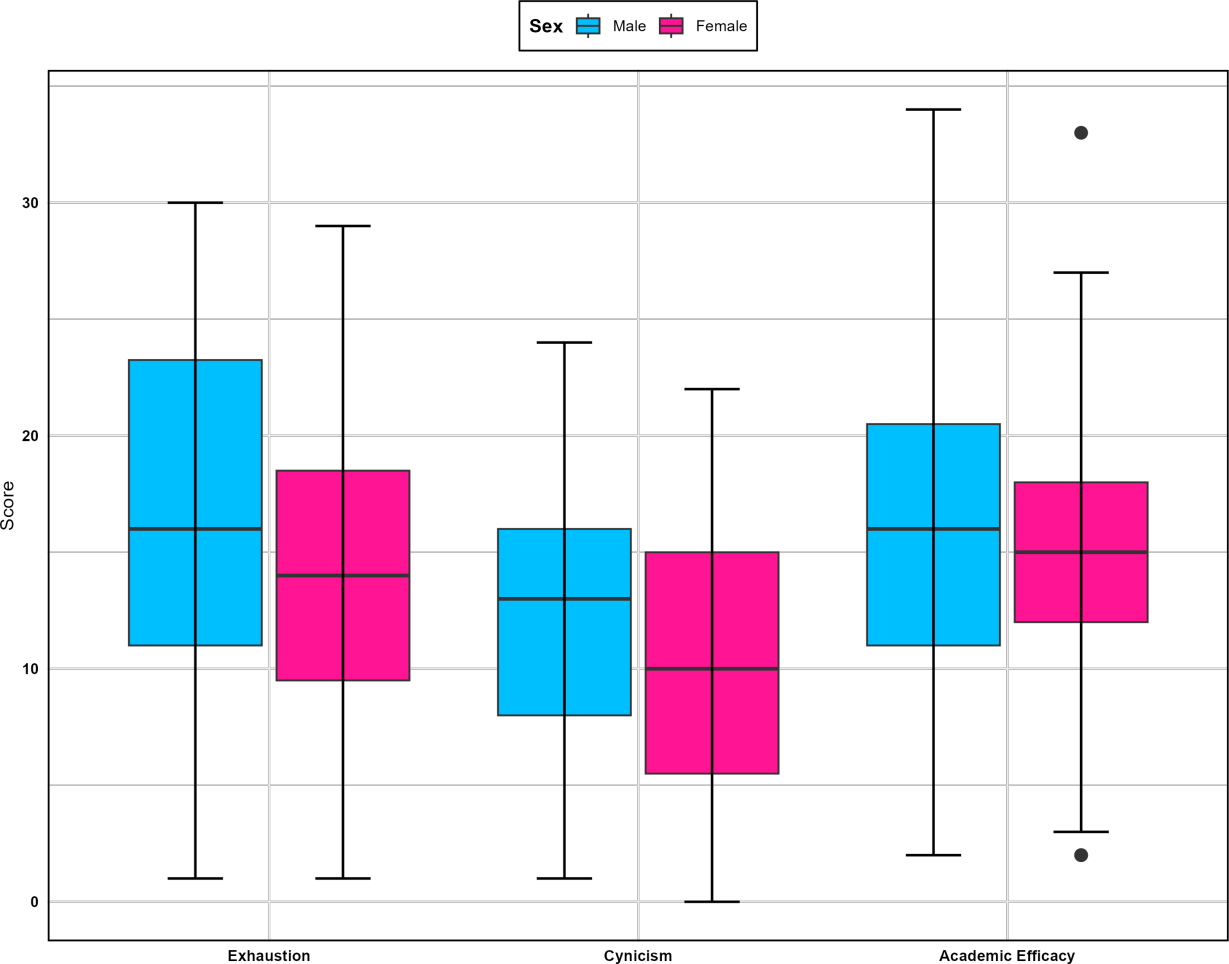
Comparison of Maslach Burnout Inventory-Student Survey subscale scores by sex.

The boxplots in Figures1  2 display the MBI-SS subscale scores across genders and academic levels. According to this figure, academic efficacy had the widest range of scores for male students. Additionally, female students exhibited lower mean scores compared to male students across all subscales. These boxplots indicate that interns had higher burnout scores overall. More detailed descriptive statistics can be found in Multimedia Appendix 1. Figure 3 shows a comparison of the total scores on the SCL-90-R between different levels, revealing that externs had the highest scores.

**Figure 2.**
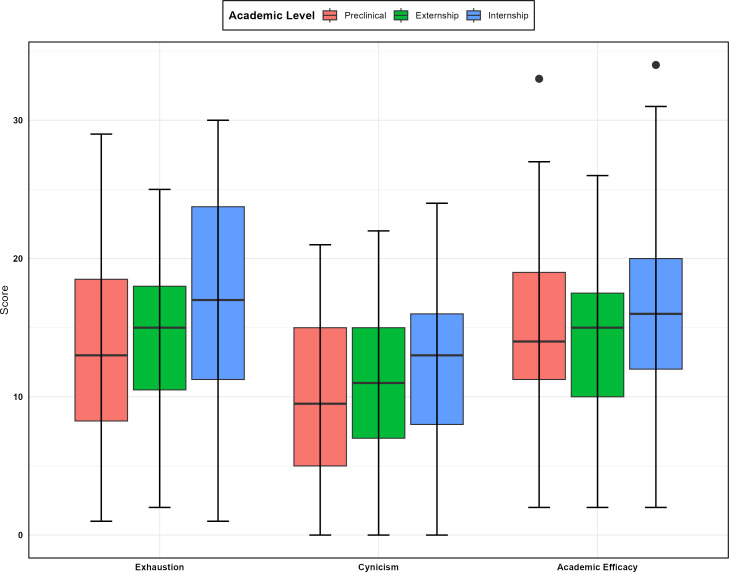
Comparison of Maslach Burnout Inventory-Student Survey subscale scores across academic levels.

**Figure 3.**
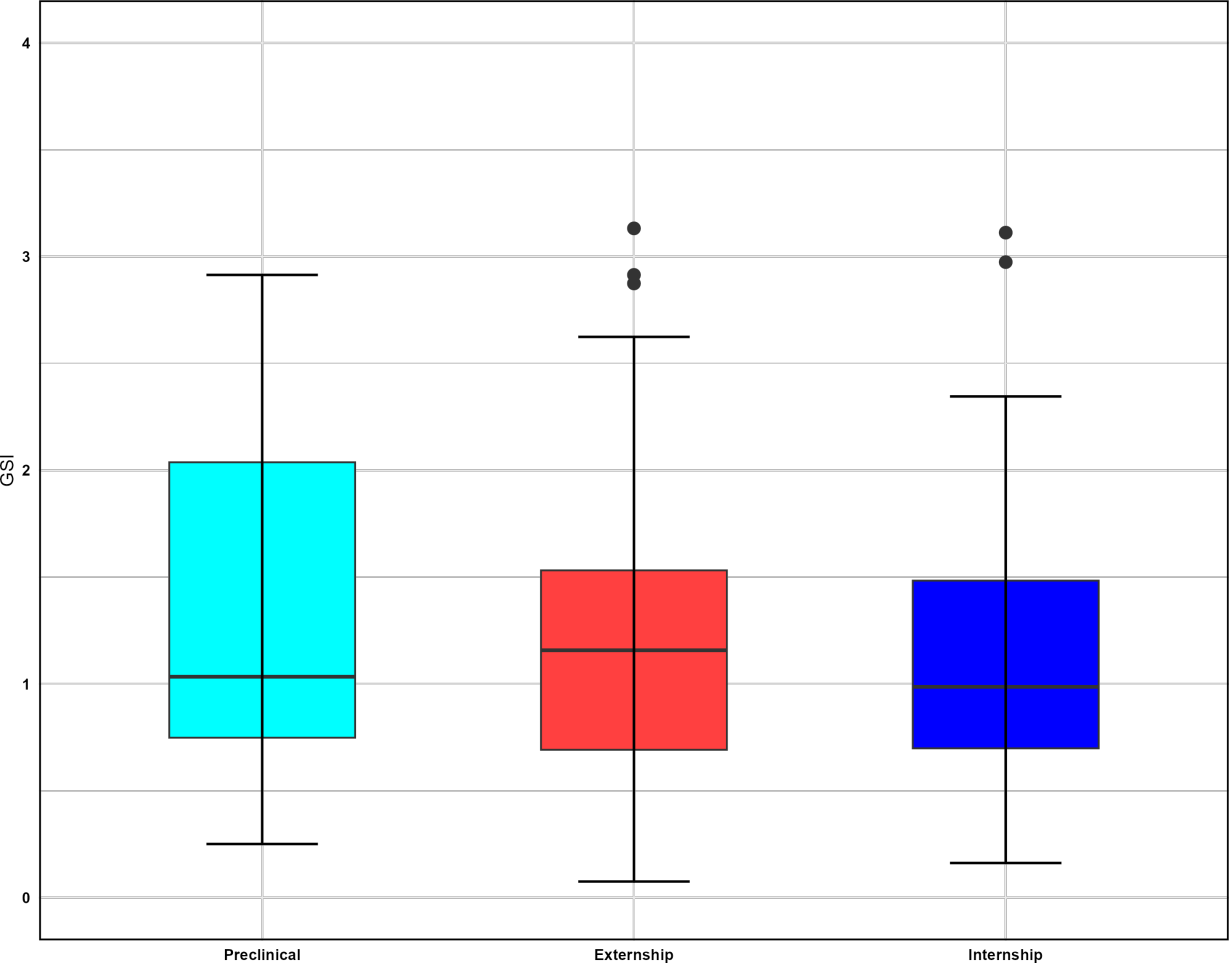
Comparison of Global Severity Index scores across academic levels.

Initially, the correlation between the three dimensions of burnout (academic efficacy, cynicism, and emotional exhaustion) was examined in the modeling data. The findings revealed that academic efficacy had a significant correlation with both cynicism (*r*=0.41, *P*<0.05) and emotional exhaustion (*r*=0.37, *P*<0.05). Additionally, emotional exhaustion positively correlated with cynicism (*r*=0.78, *P*<0.05). Given these significant correlations among the burnout dimensions, a multivariable regression analysis was deemed appropriate for further modeling. Table 3 presents the results of the multivariable regression analysis. An increase of one score in GSI corresponded to an increase of 5.67, 1.71, and 4.69 scores in emotional exhaustion, cynicism, and academic efficacy, respectively. Overall, the students in the internship phase has 4.19 and 3.02 scores higher than preclinical students in emotional exhaustion and academic efficacy, respectively, whereas they had only a 0.24-difference in cynicism. Furthermore, males scored 1.53 and 0.10 points lower than female students, respectively. A comparison of β coefficients shows that GSI and internship status had significantly different effects on the three dimensions of the MBI-SS.

**Table 3.** The association of educational burnout with mental well-being, academic level, and sex.

	Emotional exhaustion				Cynicism				Academic Efficacy				Equality of β coefficients	
	β coefficient	95% CI	*F* value	*P* value	β coefficient	95% CI	*F* value	*P* value	β coefficient	95% CI	*F* value	*P* value	*F* value	*P* value
GSI^a^	5.67	4.02-7.32	6.55	<.001	1.71	1.41-2.00	5.56	<.001	4.69	3.31-6.07	4.79	<.001	18.72	<.001
Academic levels														
Preclinical	-	-	-		-	-	-		-	-	-			
Externship	1.17	−1.83 to 4.18	0.68	.44	−0.32	−0.86 to 0.21	−1.00	.23	0.56	−1.95 to 3.08	0.52	.66	0.59	.49
Internship	4.19	0.97-7.41	2.41	.01	−0.24	−0.81 to 0.33	−1.98	.40	3.02	0.33-5.71	3.10	.03	2.01	.04
Sex														
Female	-	-	-		-	-	-		-	-	-			
Male	−1.53	−4.25 to 1.20	−1.29	.27	−0.10	−0.58 to 0.38	-1.40	.69	2.34	0.06-4.61	2.02	.04	0.99	.21
*R* ^2^	0.303				0.523				0.513				

a GSI: Global Severity Index.

## Discussion

### Principal Findings

Zanjan University, a prominent institution in Iran, attracts students from various cities. Therefore, studying its students’ mental and physical well-being can provide valuable insights into the overall condition of Iranian students.

Our study examined crucial mental health issues such as hostility, obsessive-compulsive disorder, and interpersonal sensitivity among medical students. In brief, our study did not detect any statistically significant differences in overall mental health scores between students across different academic level or sex. However, phobic anxiety was the only mental health issue that was significantly different between genders. Students at the externship level had higher GSI scores. This increase in mental health problems among the students is understandable, as it occurred during their clinical rotations in hospitals, where they were exposed to diverse patient cases and experienced various illnesses for the first time in their academic careers. Facing such novel and potentially challenging situations can reasonably be expected to take an emotional toll.

In previous studies, depression, stress, and anxiety are the three most prevalent mental health issues among medical students [7,11-13,15,26,27]. Cuttilan et al [13], who reviewed studies from Asia in their meta-analysis, showed that 30% of Middle Eastern students experienced depression. While our study found a slightly lower prevalence, the difference could be caused by the university environment, sample size, and social or climatic differences. Nonetheless, the rate of depression remains considerable. Aghajani Liasi et al [7], who studied the prevalence of burnout and mental health at one of Tehran’s universities, reported a 37% of depression among medical students. They used the Depression, Anxiety, and Stress Scale questionnaire to survey mental health. Therefore, despite being of the same nationality, the main reason for the variation between the findings of our study compared to those reported by Aghajani Liasi et al may be due to differences in the questionnaires.

Anxiety is one of the significant issues experienced by medical students. A systematic review revealed the wide range of anxiety prevalence across different countries (15.5%-70.0%) [27]. Our study found an anxiety disorder prevalence of 19% among students at Zanjan University of Medical Sciences; this places our sampled student population in the lower quartile of anxiety rates compared to the broader range reported by medical trainees across the world.

Unlike the questionnaire used in our study (ie, SCL-90-R), many previous studies on student stress have used the DASS scale and reported high rates of stress among students. For example, a meta-analysis found that 52.7% of medical students reported significant stress during training [13]. Additionally, studies by Aghajani Liasi et al [7] and Moutinho et al [28] reported stress rates of approximately 30% and 47%, respectively within their student samples, despite the differences in study populations. While these percentages vary, these studies collectively highlight that clinically significant stress is a widespread and impactful issue for many students across educational contexts [7,28]. However, our study did not directly measure student stress, which is a limitation compared to previous existing research.

Our study showed that although there was no statistically significant difference in burnout scores between male and female students, female students reported lower burnout levels in the three burnout subscales compared to male students. Additionally, students in later years of medical education reported higher burnout levels than students in the initial phases. This indicates that the interns about to graduate showed higher burnout levels, especially feeling emotionally drained.

The relationship between years of medical education and burnout levels is interesting. While some studies have suggested that burnout levels may increase with advancing years of medical education due to prolonged exposure to stressors, the evidence remains inconclusive [29,30]. This suggests that the intense pressures of medical school take an cumulative toll.

Prior studies have found that medical students experience some of the highest rates of burnout compared to other populations [4,6,11,20]. However, findings regarding the relationship between gender and burnout have been mixed [29,31-33]. There was more evidence suggesting that male students are more likely to face burnout than female students [6]. Therefore, it can be concluded that the relationship between gender and burnout in medical students may be influenced by various factors such as the specific population, sample sizes, and the definition of burnout used in the research.

Our study explored several potential influencing factors on the three burnout dimensions in medical students, including mental health status, gender, and academic level. These variables significantly impacted emotional exhaustion, cynicism, and academic efficacy scores. To date, no study had directly examined the linkage between mental health disorders and burnout in this population, representing a gap in understanding. However, related research by Dyrbye et al [16] showed associations between positive well-being and professionalism, which burnout may undermine. Additionally, psychologists have suggested that students with psychiatric conditions demonstrate greater emotional exhaustion [17,18,34]. Notably, in our analysis, mental health had a much more significant effect on emotional exhaustion compared to the other burnout facets. Other studies found students with higher burnout reported more suicidal thoughts and behaviors [6,17,18,26,27,34,35]. Integrating those findings with our results suggests that mental health could play an intermediary role between burnout and suicidal risks. These interrelationships between wellness, distress, and functioning highlight the need for more holistic support to promote student resilience.

### Limitations

This study has some limitations. The cross-sectional design cannot determine causal relationships between variables. Additionally, the convenience sampling and voluntary participation could indicate that students with psychological issues may have been less inclined to take part or answer honestly. While different variables were recorded, others such as physical activity, social support, and economic status, should be investigated in future studies. Longitudinal follow-up studies warrant a better understanding of mental health’s impact on burnout trajectories.

Another limitation was the rate of female participation compared to male participants for two reasons. According to the university’s annual statistics, about 55% of students are women, increasing the female sample rate in the convenience sampling method. However, among the Iranian population, women are more interested in psychological issues and experience exhaustion about improving mental health, resulting in more women participation in our study.

An additional limitation is the potential link between financial issues, mental health, and burnout. Our survey did not include detailed questions about participants’ financial situations, which could have influenced their responses to other questions. To partially address this, we included a question about place of residence, which could indirectly reflect financial circumstances and their possible effects on other survey responses.

### Conclusion

The demanding nature of academic work and personal lives faced by medical students can take a severe mental toll, leading to burnout. Despite being educated on physical and psychological health, students often neglect their own well-being. This research confirms that mental health issues directly contribute to students’ emotional exhaustion, cynicism, and reduced feelings of academic self-efficacy. Both burnout and psychological problems increase the risk of students dropping out or deciding against careers as general practitioners after graduation, resulting in wasted resources invested in their training. Most alarmingly, if the society cannot ensure the mental well-being of its future doctors, the overall population’s health will suffer consequences. There is an undeniable connection between medical trainees’ health and the communities they will serve. Fostering resilience and coping abilities in students must be a key priority, as their personal health and capacity to provide quality patient care in the future hinges on it. The findings of this study highlight the prevalence of burnout and mental health issues among medical students, underscoring the profound importance of addressing this problem for the well-being of the general population, who will rely on these future physicians for care.

## Supplementary material

10.2196/58008 Multimedia Appendix 1 Descriptive statistics of exhaustion, cynicism, academic efficacy across sex and academic level.

## Abbreviations

GSI: Global Severity Index
MBI-SS: Maslach Burnout Inventory-Student Survey
SCL-90: Symptom Checklist-90
SCL-90-R: Symptom Checklist-90-Revised
