# Supplementary material for: Exploring Connections Between Mental Health, Burnout, and Academic Factors Among Medical Students at an Iranian University: Cross-Sectional Questionnaire Study
Source: JMIR Med Educ. 2025 May 15;11:e58008. doi: 10.2196/58008 (PMC12097282; doi:10.2196/58008)
Supplement: Multimedia Appendix 1 [file mededu-v11-e58008-s001.docx]

**Appendix 1**

| Descriptive statistics of exhaustion, cynicism, academic efficacy across sex and academic level | | | | | | | | |
| --- | --- | --- | --- | --- | --- | --- | --- | --- |
|  |  |  | Count | Mean | SD | Median | Q_1_ | Q_3_ |
| Exhaustion | Sex | Male | 44 | 16.66 | 7.98 | 16.0 | 11.0 | 23.5 |
|  |  | Female | 87 | 14.16 | 6.47 | 14.0 | 9.0 | 19.0 |
|  | level | Preclinical | 42 | 13.76 | 7.13 | 13.0 | 8.0 | 19.0 |
|  |  | Externship | 47 | 14.45 | 5.92 | 15.0 | 10.0 | 18.0 |
|  |  | Internship | 42 | 16.86 | 7.97 | 17.0 | 11.0 | 24.0 |
| Cynicism | Sex | Male | 44 | 12.25 | 5.86 | 13.0 | 8.0 | 16.0 |
|  |  | Female | 87 | 10.14 | 5.82 | 10.0 | 5.0 | 15.0 |
|  | level | Preclinical | 42 | 9.74 | 6.28 | 9.5 | 5.0 | 15.0 |
|  |  | Externship | 47 | 10.77 | 5.53 | 11.0 | 6.0 | 15.0 |
|  |  | Internship | 42 | 12.05 | 5.81 | 13.0 | 8.0 | 16.0 |
| Academic Efficacy | Sex | Male | 44 | 15.93 | 7.5 | 16.0 | 11.0 | 21.0 |
|  |  | Female | 87 | 14.51 | 5.58 | 15.0 | 12.0 | 18.0 |
|  | level | Preclinical | 42 | 14.74 | 6.64 | 14.0 | 11.0 | 19.0 |
|  |  | Externship | 47 | 13.91 | 5.7 | 15.0 | 9.0 | 18.0 |
|  |  | Internship | 42 | 16.43 | 6.45 | 16.0 | 12.0 | 20.0 |
